# Understanding knowledge and approval for sociopolitical groups: results from the 2023 National Survey of Gun Policy

**DOI:** 10.1186/s40621-025-00575-z

**Published:** 2025-04-10

**Authors:** Rebecca Valek, Julie A. Ward, Vanya Jones, Tim Carey, Cassandra K. Crifasi

**Affiliations:** 1 Center for Gun Violence Solutions, Department of Health Policy and Management, Johns Hopkins Bloomberg School of Public Health, Baltimore, MD USA; 2 School of Public Health, Oregon Health & Science University-Portland State University, Portland, OR USA; 3 Department of Medicine, Health, and Society, Vanderbilt College of Arts and Science, Vanderbilt University, Nashville, TN USA; 4 Department of Health, Behavior and Society, Johns Hopkins Bloomberg School of Public Health, Baltimore, MD USA

**Keywords:** Gun ownership, Sociopolitical movements, Political violence

## Abstract

**Background:**

Increased concerns of political violence in the US have drawn attention to sociopolitical movements across the political spectrum. The 2023 National Survey of Gun Policy sought to characterize approval of these movements and whether gun ownership was associated with this approval.

**Methods:**

The National Survey of Gun Policy was fielded from 1/4/23 − 2/6/23 among a nationally representative sample of US adults (*N* = 3,096), including gun owners (*n* = 1,002). Respondents rated their level of approval for the militia, antifascist (Antifa), white supremacy, Christian nationalist, boogaloo, and anarchist movements. Logistic regression was used to compare differences in movement approval by gun ownership.

**Results:**

Approval of each movement was relatively low, ranging from 4% for the boogaloo movement to 13% for the Christian nationalist movement. Proportions of respondents that reported lacking knowledge was highest for the boogaloo movement (64%) and lowest for the white supremacy movement (17%); these two movements had similar proportions of approval (4% and 5%, respectively). Significantly larger proportions of gun owners reported both knowledge and approval of any of the six movements compared to non-gun owners, but differences in approval by gun ownership were no longer significant when only comparing those with knowledge of the movements.

**Conclusions:**

Results indicate low probabilities of knowledge and approval. Moreover, greater knowledge was not accompanied by greater approval (e.g., white supremacy). Gun ownership was associated with movement knowledge, but not with movement approval among those with knowledge. These findings suggest opportunities for more proactive public health messaging to appeal to majority groups to resist movements that may sow division.

**Supplementary Information:**

The online version contains supplementary material available at 10.1186/s40621-025-00575-z.

## Background

Threats and concerns of political violence have grown across the United States in recent years, concurrent with a firearm purchasing surge and increased political extremism [1]. In one national survey conducted in 2022, 13% of respondents reported agreeing strongly or very strongly that “in the next few years, there will be a civil war in the United States” [2]. This proportion decreased to 5.7% in 2023, but the perceived expectation of personal firearm use in future political violence situations increased among those who supported political violence for certain objectives [3]. Additionally, authors warned of a potential increase in support for political violence in 2024 given the presidential election [3]. Another 2022 survey reported that 23% of Americans agree with the use of violence to “save our country,” including 33% of Republicans and 13% of Democrats [4]. Data from the Center for Strategic and International Studies show that this rise is not limited to beliefs, as the number of terrorist attacks and plots has also risen in recent years [5]. Additionally, the Capitol Police have reported record-high numbers of threats against members of Congress [6]. 

Although the majority of incidents of political violence are carried out by individuals rather than organized groups, the growth in violence and extremism have drawn attention to organized movements across the political spectrum [7]. According to the Center for Strategic and International Studies, right-wing terrorism “refers to the use or threat of violence by sub-national or non-state entities whose goals may include racial or ethnic supremacy; opposition to government authority; anger at women…; and outrage against certain policies, such as abortion” [5]. Individuals with right-wing ideologies have perpetrated most terrorist attacks and plots in the U.S. from 1994 to 2020 and a larger proportion of all attacks in recent years [5]. Some of these groups classified as having right-wing ideologies include militia groups, which may vary broadly between groups, but commonly share anti-government and pro-second amendment views; [8] white supremacy groups, which are characterized by a decentralized network of groups and a belief in the superiority of the white race; [9] Christian nationalist groups, which are characterized by the belief that the U.S. was founded as, and should continue to be, a Christian nation by “idealiz[ing] and advocat[ing for] a fusion of American civic life with a particular type of Christian identity and culture;” [10–12] and the Boogaloo movement, which is not a group itself, but rather comprised of a variety of supporters connected by antigovernment and anti-law enforcement ideology and support for a civil war [13, 14]. 

In 2023, far-right militia groups were involved in 91% of all U.S demonstrations that were violent or turned violent [15]. In the same year, white nationalist groups held nearly 150 combat training events [15]. In 2022, white supremacy and white nationalism were the primary factors driving far-right protests, accounting for about 21% of far-right demonstrations [16]. A Federal Bureau of Investigations and Department of Homeland Security assessment stated that, in 2021, racially or ethnically motivated violent extremists “advocating the superiority of the white race and anti-authority or anti-government violent extremists, specifically militia violent extremists, presented the most lethal threat categories” [17]. The presence of firearms at these protests and demonstrations is a known risk factor for demonstrations becoming violent or even deadly, with armed protests being nearly six times as likely to become violent compared to unarmed demonstrations [18]. 

In addition to these right-leaning groups, left-wing groups include anarchists, who are “fundamentally opposed to a centralized government and capitalism,” and Antifa, or the anti-fascist movement [5]. Antifa is comprised of a “decentralized network of far-left militants that oppose what they believe are fascist, racist, or otherwise right-wing extremists,” with roots in communism, anarchism, and socialism [5, 19]. Grouped by the Federal Bureau of Investigation and the Department of Homeland Security as “anarchist violent extremists”, these groups are occasionally responsible for violent assaults but primarily engage in property crimes [17]. 

In a national survey on political violence, respondents who approved of right-wing organizations like those described above more frequently agreed that violence is usually or always justified to advance political objectives, reported that they were personally willing to engage in such violence, and believed that it was very or extremely likely that they would be armed in a future political violence situation compared to those who did not report approval of these movements [20]. This expectation of being armed in future political violence situations and willingness to engage in political violence is concerning given that the presence of firearms at demonstrations increases the risk of violence, many states have relaxed or removed requirements to obtain a license before carrying guns in public spaces, and there remains open debate regarding locations where guns can or cannot be legally carried [18, 21]. Additionally, research on the gun purchasing surge during the COVID-19 pandemic found that those who purchased a firearm during the pandemic were significantly more likely than non-gun owners and pre-existing gun owners who did not purchase a firearm during the pandemic to endorse conspiracy theories, distrust the government, engage in protest activity, and support political violence [22]. 

Given the concern about the potential role of these social movements or ideologies in violence, understanding support for these political and social movements is necessary, particularly among gun owners as this group continues to grow and diversify. The 2023 National Survey of Gun Policy sought to characterize approval of these movements and understand whether gun ownership was associated with this approval.

## Methods

We fielded the 2023 National Survey of Gun Policy using NORC’s AmeriSpeak panel from January 4 to February 6, 2023. The AmeriSpeak Panel is drawn from the NORC National Frame, a nationally representative, probability-based panel of adults ages 18 and older that uses address-based sampling to cover 97% of U.S. households [23, 24]. Interviews were administered online and by phone in both English and Spanish. The survey completion rate was 76.5%, for a final sample of 3,096 respondents. We oversampled for gun owners (*n* = 1,002). For additional details on the survey methods, see [25, 26]. 

Gun ownership was determined through two questions: “Do you happen to have in your home or garage any guns or revolvers?” and “Do any of these guns personally belong to you?” A gun owner was defined as a respondent who was the personal owner of at least one firearm.

Respondents were also asked to rank their approval for six different social movements: the militia movement, the antifascist (Antifa) movement, the white supremacy movement, the Christian nationalist movement, the boogaloo movement, and the anarchist movement. Options ranged from ‘do not approve’ to ‘very strongly approve’ on a four-point Likert scale, with additional options to select ‘I don’t know enough about this political or social movement to rate it’ or ‘I have never heard of this political or social movement’. We grouped these responses into three categories: ‘approve’, consisting of those who selected ‘somewhat approve’, ‘strongly approve’, and ‘very strongly approve’; ‘do not approve’; and ‘lack of knowledge’, consisting of those who selected ‘I don’t know enough about this political or social movement to rate it’ or ‘I have never heard of this political or social movement’. We created dichotomous variables for each response option to enable comparison of approval and knowledge by gun ownership. As a sensitivity analysis, we also examined approval among only respondents that reported knowledge of the movements, comparing those who selected ‘somewhat approve’, ‘strongly approve’, and ‘very strongly approve’ to those who selected ‘do not approve’. Additionally, we examined knowledge and approval of any movement by creating dichotomous variables indicating whether each respondent indicated approval of any or lack of knowledge of all of the six sociopolitical movements.

Survey-weighted logistic regression was used to compare differences in movement knowledge and approval between gun owners and non-gun owners. We conducted analyses using survey weights provided by NORC to adjust for known sampling deviations and survey nonresponse and to ensure the sample was representative of the U.S. population (Additional file 1–Supplemental Table 1) [27–30]. Results are presented as unadjusted weighted proportions with 95% confidence intervals. As a sensitivity analysis, we also calculated the average predicted probabilities of movement knowledge and approval to assess whether differences by gun ownership remained after accounting for political party affiliation and demographic characteristics such as race, sex, age, income, educational attainment, employment status, and living in a metropolitan area. All analyses were conducted using the *svy* command in Stata version 17.0. This study was reviewed and approved by the Johns Hopkins Bloomberg School of Public Health institutional review board.

## Results

**Fig. 1:**
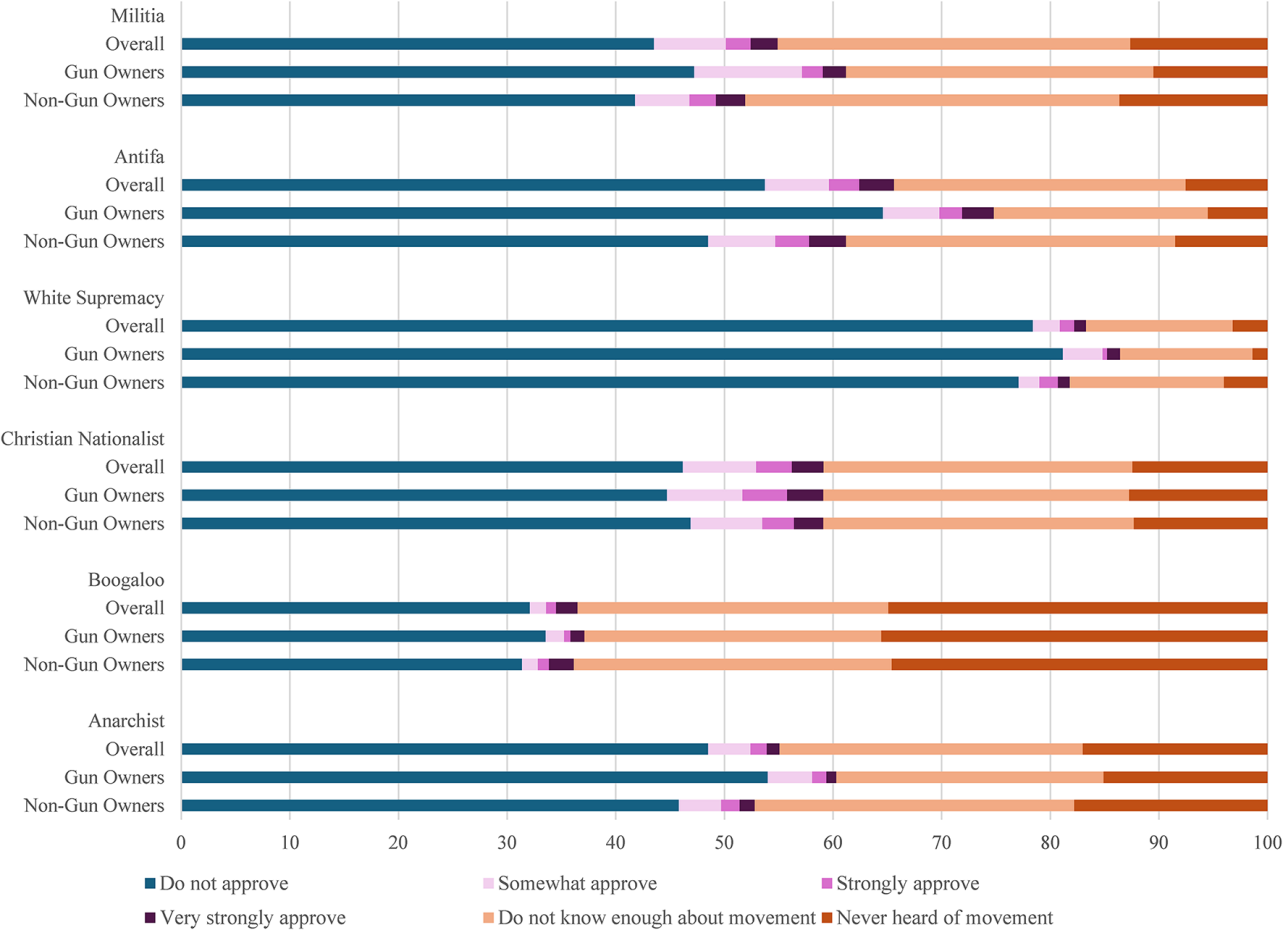
Weighted proportions of knowledge and approval of sociopolitical movements by gun ownership

Overall, reported approval of the movements was relatively low: 4% for the boogaloo movement, 5% for the white supremacy movement, 7% for the anarchist movement, 11% for the militia movement, 12% for the Antifa movement, and 13% for the Christian nationalist movement **(**Table 1**)**. Among those that did express approval of the six movements, most selected only “somewhat approve,” with much smaller proportions of respondents approving strongly or very strongly **(**Fig. 1**)**. Approximately a quarter of respondents expressed approval of at least one of the six sociopolitical movements (27%; 95% CI: 25.1–29.5).

**Table 1:** Weighted proportions of approval, disapproval, and knowledge of sociopolitical movements by gun ownership

		Overall % (CI) (N = 3,096)	Gun ownership	
			Gun owner % (CI) **(n = 1**,**002)**	Non-gun owner % (CI) **(n = 2**,**094)**
**The militia movement**				
Approve		11.4 (9.9–13.1)	14.0 (11.5–17.0)	10.2* **(8.3–12.3)**
Do not approve		43.5 (41.2–45.9)	47.2 (43.3–51.2)	41.8* **(38.8–44.8)**
Lack of knowledge about movement		45.1 (42.6–47.5)	38.8 (35.0–42.8)	48.1* **(45.0–51.2)**
**The antifascist (Antifa) movement**				
Approve		11.9 (10.4–13.6)	10.2 (8.2–12.6)	12.7 (10.7–15.0)
Do not approve		53.7 (51.3–56.2)	64.6 (60.7–68.4)	48.5* **(45.5–51.6)**
Lack of knowledge about movement		34.4 (32.0–36.8)	25.2 (21.7–29.0)	38.8* **(35.8–41.8)**
**The white supremacy movement**				
Approve		4.9 (3.9–6.2)	5.3 (3.7–7.4)	4.7 (3.5–6.4)
Do not approve		78.4 (76.2–80.5)	81.2 (77.5–84.3)	77.1 (74.3–79.7)
Lack of knowledge about movement		16.7 (14.8–18.7)	13.6 (10.8–17.0)	18.2* **(15.8–20.8)**
**The Christian nationalist movement**				
Approve		13.0 (11.4–14.7)	14.4 (11.9–17.2)	12.3 (10.3–14.6)
Do not approve		46.2 (43.8–48.6)	44.7 (40.9–48.7)	46.9 (43.8–50.0)
Lack of knowledge about movement		40.9 (38.5–43.3)	40.9 (37.0–44.9)	40.1 (37.9–44.0)
**The boogaloo movement**				
Approve		4.4 (3.4–5.6)	3.6 (2.4–5.3)	4.8 (3.5–6.5)
Do not approve		32.1 (29.9–34.3)	33.6 (30.0–37.3)	31.4 (28.7–34.2)
Lack of knowledge about movement		63.5 (61.2–65.8)	62.9 (59.1–66.5)	63.9 (60.9–66.7)
**The anarchist movement**				
Approve		6.7 (5.6–8.1)	6.3 (4.7–8.4)	6.9 (5.5–8.8)
Do not approve		48.5 (46.0–50.9)	54.0 (50.0–57.9)	45.8* **(42.8–48.9)**
Lack of knowledge about movement		44.8 (42.4–47.3)	39.7 (35.9–43.7)	47.3* **(44.3–50.4)**
**Collective knowledge and approval**				
Approval of at least 1 of the 6 movements		27.3 (25.1–29.5)	30.6 (27.1–34.3)	25.7* **(23.1–28.5)**
Lack of knowledge about all 6 movements		11.7 (10.1–13.5)	8.8 (6.5–11.8)	13.1* **(11.1–15.5)**

*P-values ≤ 0.05 were considered significant (shown in bold)

Weighted proportions of respondents lacking knowledge or reporting disapproval were similar for the militia (lack knowledge: 45%; disapprove: 44%), Christian nationalist (lack knowledge: 41%; disapprove: 46%), and anarchist (lack knowledge: 45%; disapprove: 49%) movements. There were wider gaps between knowledge and disapproval for the Antifa movement (lack knowledge: 34%; disapprove: 54%) and the boogaloo movement (lack knowledge: 64%; disapprove: 32%). The proportion of respondents that reported lacking knowledge of the white supremacy movement was the smallest (17%), with 78% reporting disapproval and 5% reporting approval. 12% of respondents reported lacking knowledge of all six movements.

Differences in weighted proportions of movement knowledge and approval were observed between gun owners (*n* = 1,002) and non-gun owners (*n* = 2,094) for four of the six movements, with no differences by gun ownership observed for the Christian nationalist or the boogaloo movements **(**Table 1**)**. A significantly larger proportion of gun owners approved of the militia movement (14%; 95% CI: 11.5–17.0) compared to non-gun owners (10%; 95% CI: 8.3–12.3). Significantly larger proportions of gun owners expressed disapproval of the militia movement (47%; 95% CI: 43.3–51.2), the Antifa movement (65%; 95% CI: 60.7–68.4), and the anarchist movement (54%; 95% CI: 50.0–57.9) compared to non-gun owners (militia: 42%, 95% CI: 38.8–44.8; Antifa: 49%, 95% CI: 45.5–51.6; anarchist: 46%, 95% CI: 42.8–48.9). Significantly smaller proportions of gun owners reported lacking knowledge of the militia movement (39%; 95% CI: 35.0–42.8), the Antifa movement (25%; 95% CI: 21.7–29.0), the white supremacy movement (14%; 95% CI: 10.8–17.0), and the anarchist movement (40%; 95% CI: 35.9–43.7) compared to non-gun owners (militia: 48%, 95% CI: 45.0–51.2; Antifa: 39%, 95% CI: 35.8–41.8; white supremacy: 18%, 95% CI: 15.8–20.8; anarchist: 47%, 95% CI: 44.3–50.4). When examining approval and knowledge collectively, a significantly larger proportion of gun owners reported approving of any of the six movements (31%; 95% CI: 27.1–34.3) compared to non-gun owners (26%; 95% CI: 23.1–28.5). Fewer gun owners reported lacking knowledge of all six movements (9%; 95% CI: 6.5–11.8) compared to non-gun owners (13%; 95% CI: 11.1–15.5).

When examining approval among only those who reported knowledge of the movements (Additional file 1–Supplemental Table 2), the only significant difference between gun owners and non-gun owners was for the Antifa movement, with a significantly smaller proportion of gun owners with knowledge of the Antifa movement reporting approval (14%; 95% CI: 11.0–16.8) compared to non-gun owners (21%; 95% CI: 17.7–24.2). No significant difference was observed in the collective approval of any of the six movements between gun owners (34%; 95% CI: 29.9–37.6) and non-gun owners (30%; 95% CI: 27.0–33.1) when only considering respondents who expressed knowledge of at least one movement.

When examining predicted probabilities of movement approval, disapproval, and lack of knowledge, after accounting for political party affiliation and other demographic characteristics, fewer significant differences were observed between gun owners and non-gun owners (Additional file 1–Supplemental Table 3). Only one significant difference remained: the probability of lacking knowledge of the Antifa movement was significantly higher for non-gun owners (36%; 95% CI: 33.1–38.6) than gun owners (31%; 95% CI: 26.7–34.9). Overall, weighted proportions and predicted probabilities were similar, but gun owners’ probabilities of movement approval and lack of knowledge tended to be slightly higher, and their probabilities of movement disapproval slightly lower, compared to their corresponding weighted proportions. The opposite patterns were observed among non-gun owners.

## Discussion

Generally low levels of knowledge or support for the militia movement, the antifascist (Antifa) movement, the white supremacy movement, the Christian nationalist movement, the boogaloo movement, and the anarchist movement suggest these movements or ideologies are not mainstream. Overall approval of the movements was relatively low, ranging from 4% for the boogaloo movement to 13% for the Christian nationalist movement. While support was relatively low for each movement individually, that over a quarter of respondents reported approval of at least one movement may raise concerns. When examining movement support and knowledge collectively, larger proportions of gun owners reported both knowledge and approval of at least one movement compared to non-gun owners. However, when examining approval of at least one movement among only those who reported knowledge of at least one movement, gun owners and non-gun owners expressed similar levels of approval. Additionally, when examining weighted proportions of approval, disapproval, and lacking knowledge of each movement individually among the full sample of respondents, the only significant difference in approval observed was for the militia movement. Regarding the militia movement, a larger proportion of gun owners expressed approval than non-gun owners. However, a larger proportion of gun owners than non-gun owners also expressed disapproval for the militia movement, with the bigger difference between gun owners and non-gun owners being in reported knowledge of this movement rather than approval. Disapproval was higher among gun owners than non-gun owners for the Antifa and anarchist movements as well. Moreover, greater knowledge of the white supremacy movement was not accompanied by higher levels of support.

Evidence that movements like the white supremacy movement are more widely known, but remain broadly unpopular, highlights an opportunity for public health messaging targeting the majority about gun violence prevention and extremism, as well as an opportunity to tailor public health and violence prevention messages and programs to people participating in these extreme groups. In recent years, protests and demonstrations involving these movements have sparked increased media coverage. In the wake of these incidents, Google searches spiked as the public searched for more information on these incidents and movements [31, 32]. Still, while many Americans appear to know about the white supremacy movement, for example, the messages and ideas of this group may remain unpopular, even as knowledge of the group grows. The type of knowledge or messaging about these movements may matter. Media promoting radicalizing information and extremist messaging has been associated with support for violent extremism, potentially supporting movement recruitment [33]. By prioritizing responsible media practices, newsrooms and media agencies may instead aid in countering extremist violence and narratives [33, 34]. Research on perceptions of media reporting among extremists has suggested ways to prioritize responsible media practices, emphasizing the importance of “factual, accurate, objective, and unemotional news coverage” [35]. Additionally, disseminating messages from trusted leaders denouncing violence has been associated with reductions in support for partisan violence, particularly among those most likely to support political violence [36]. Anti-violence messaging targeting supporters of these various political and social movements may discourage such violence or reduce the risk that demonstrations become violent.

Additionally, these findings further highlight the diversity of viewpoints among gun owners, suggesting that these movements may leverage gun ownership to achieve their aims instead of gun ownership being a gateway to certain ideologies. While significant differences were observed between gun owners and non-gun owners, these differences were primarily in reports of lacking knowledge and disapproval; differences in approval were only observed for the militia movement. Additionally, when accounting for political party affiliation and other demographic variables, few differences were observed in the predicted probabilities of knowledge and approval by gun ownership, suggesting that these other factors may be influencing the relationship between gun ownership and movement knowledge and approval. Future research should explore other potential drivers of movement support, as well as how policy support varies across supporters of these social movements.

These findings should be considered in the context of various limitations. Sampling biases may have impacted our findings, but these biases are minimized through NORC at the University of Chicago’s probability-based sampling, which covers 95% or more of U.S. households [24]. Social desirability bias may have also impacted responses, but the use of an anonymous survey minimized this concern. Additionally, people own guns for many reasons, including protection and recreation, and firearm purchasing may be motivated by a variety of factors, including racial resentment and concerns of racial or political violence [25, 26, 37]. Future research should examine within-group differences in group support by reasons for gun ownership or other demographics or affiliations. Lastly, this survey was conducted in January of 2023 and results may not be generalizable across time. Responses may shift with changing political discourse, administrations, or cultural norms. Future research should explore changes in knowledge and approval of these movements over time and in response to various events and contextual factors.

## Conclusion

Approval of militia, antifa, white supremacy, Christian nationalist, boogaloo, and anarchist movements was low among both gun owners and non-gunowners in this nationally representative survey. Understanding the drivers of support for these movements is essential to reducing engagement with these movements and increasing safety. Our findings suggest that gun ownership is associated with knowledge of the movements, but, among those with knowledge, gun ownership is not associated with movement approval, nor is knowledge indicative of movement approval. While vocal groups may wield influence, these findings suggest an opportunity for more proactive public health messaging to appeal to majority groups to resist movements that sow division.

## Electronic supplementary materials

Additional file 1: Supplemental Data Table 1, Supplemental Data Table 2, and Supplemental Data Table 3: Description of data: Additional File 1 includes Supplemental Data Table 1, which displays the weighted and unweighted demographic characteristics of the study sample and national rates; Supplemental Data Table 2, which shows the weighted proportions of movement approval among those with knowledge of the movements by gun ownership; and Supplemental Data Table 3, which depicts the weighted predicted probabilities of approval, disapproval, and knowledge of each movement by gun ownership.

## Data Availability

The datasets generated and/or analyzed during the current study are not publicly available as analyses are ongoing, but the datasets will be made available to qualified researchers subject to the terms of a data use agreement.
