# Supplementary material for: Understanding knowledge and approval for sociopolitical groups: results from the 2023 National Survey of Gun Policy
Source: Inj Epidemiol. 2025 Apr 10;12:20. doi: 10.1186/s40621-025-00575-z (PMC11984245; doi:10.1186/s40621-025-00575-z)
Supplement: Supplementary file 1 — Additional file 1: Supplemental Data Table 1, Supplemental Data Table 2, and Supplemental Data Table 3: Description of data: Additional File 1 includes Supplemental Data Table 1, which displays the weighted and unweighted demographic characteristics of the study sample and national rates; Supplemental Data Table 2, which shows the weighted proportions of movement approval among those with knowledge of the movements by gun ownership; and Supplemental Data Table 3, which depicts the weighted predicted probabilities of approval, disapproval, and knowledge of each movement by gun ownership. [file 40621_2025_575_MOESM1_ESM.pdf]

Additional File 1: Supplemental Data Table

Supplemental Table 1: Weighted and unweighted demographic characteristics of the study sample and national rates

		National Comparison (%)	Unweighted (%)	Weighted (%)
Female		51.0	46.9	49.7
Age				
	18-34	29.1	23.8	28.5
	35-49	24.6	25.3	23.8
	50-64	24.7	26.5	25.1
	65+	21.6	24.5	22.6
Race/Ethnicity				
	White, non-Hispanic	60.8	44.9	62.7
	Black, non-Hispanic	11.7	21.6	12.0
	Asian, non-Hispanic	6.1	10.9	6.3
	Other or multi-racial, non-Hispanic	4.6	2.1	2.2
	Hispanic	16.8	20.5	16.9
Education				
	High school diploma or less	38.0	22.1	37.8
	Some college	29.5	39.5	26.7
	Bachelor's degree or higher	32.4	38.4	35.5
Household income				
	<$35,000	25.2	28.1	28.8
	$35,000-$74,999	28.1	30.7	29.6
	>$75,000	46.8	41.2	41.6
Employment status				
	Employed	61.3	60.5	58.7
	Unemployed	2.3	6.3	6.8
	Other	36.5	33.2	34.5
Region				
	Northeast	17.6	13.0	17.2
	Midwest	20.7	23.8	20.7
	South	38.1	37.7	38.9

	West	23.6	25.5	23.2
Political Party Affiliation				
	Republican	31.5	23.6	28.5
	Independent	33.8	37.6	38.6
	Democrat	34.7	38.7	32.8

NOTE: National comparison data were obtained for the U.S. population ages 18+ from the American Community Survey (ACS) 1-year estimates for 2021, the 2020 Decennial Census, the 2022 Current Population Survey, and the American National Election Studies (ANES).[27-30]

Supplemental Table 2: Weighted proportions of movement approval among those with knowledge of the movements, by gun ownership

		Overall % (CI) (N=3,096)	Gun Ownership	
			Gun owner % (CI) (n=1,002)	Non-gun owner % (CI) (n=2,094)
Militia movement				
	Approve	20.7 (18.2-23.6)	22.8 (18.9-27.3)	19.6 (16.3-23.3)
The antifascist (Antifa) movement				
	Approve	18.1 (15.9-20.6)	13.6 (11.0-16.8)	**20.8*** **(17.7-24.2)**
The white supremacy movement				
	Approve	5.9 (4.7-7.4)	6.1 (4.3-8.5)	5.8 (4.3-7.8)
The Christian nationalist movement				
	Approve	21.9 (19.4-24.7)	24.3 (20.4-28.8)	20.7 (17.6-24.3)
The boogaloo movement				
	Approve	12.0 (9.4-15.2)	9.6 (6.6-13.9)	13.2 (9.8-17.6)
The anarchist movement				
	Approve	12.2 (10.1-14.6)	10.4 (7.8-13.8)	13.2 (10.4-16.5)
Collective approval				
	Approval of at least 1 of the 6 movements	31.2 (28.8-33.6)	33.7 (29.9-37.6)	29.9 (27.0-33.1)

*P-values ≤ 0.05 were considered significant (shown in bold).

Supplemental Table 3: Weighted predicted probabilities of approval, disapproval, and knowledge of movements, by gun ownership

		Overall % (CI) (N=3,096)	Gun Ownership	
			Gun owner % (CI) (n=1,002)	Non-gun owner % (CI) (n=2,094)
Militia movement				
	Approve	11.4 (9.8-12.9)	13.2 (10.4-16.1)	10.4 (8.5-12.4)
	Do not approve	43.5 (41.4-45.7)	43.6 (39.8-47.4)	43.5 (40.8-46.2)
	Lack of knowledge about movement	45.1 (42.7-47.4)	43.3 (39.1-47.5)	45.9 (43.0-48.8)
The antifascist (Antifa) movement				
	Approve	11.9 (10.3-13.5)	11.6 (9.0-14.2)	12.0 (10.0-14.0)
	Do not approve	53.7 (51.5-56.0)	57.2 (53.1-61.3)	52.2 (49.3-55.0)
	Lack of knowledge about movement	34.4 (32.2-36.6)	30.8 (26.7-34.9)	**35.9* (33.1-38.6)**
The white supremacy movement				
	Approve	4.9 (3.8-6.0)	5.8 (3.7-8.0)	4.5 (3.2-5.9)
	Do not approve	78.4 (76.4-80.4)	78.8 (75.2-82.5)	78.2 (75.7-80.7)
	Lack of knowledge about movement	16.7 (14.8-18.6)	15.4 (12.0-18.7)	17.3 (15.0-19.5)
The Christian nationalist movement				
	Approve	12.9 (11.3-14.6)	13.7 (10.9-16.5)	12.6 (10.4-14.7)
	Do not approve	46.2	44.2	47.1

		(43.9-48.5)	(40.3-48.2)	(44.3-50.0)
	Lack of knowledge about movement	40.9<br>(38.5-43.2)	42.4<br>(38.3-46.5)	40.1<br>(37.2-43.1)
The boogaloo movement				
	Approve	4.4<br>(3.3-5.5)	4.5<br>(2.6-6.4)	4.4<br>(3.0-5.7)
	Do not approve	32.1<br>(30.0-34.2)	30.9<br>(27.2-34.5)	32.7<br>(30.0-35.4)
	Lack of knowledge about movement	63.5<br>(61.3-65.8)	64.9<br>(61.0-68.7)	62.9<br>(60.0-65.7)
The anarchist movement				
	Approve	6.7<br>(5.5-8.0)	7.5<br>(5.2-9.8)	6.4<br>(4.9-8.0)
	Do not approve	48.5<br>(46.2-50.8)	48.7<br>(44.7-52.7)	48.3<br>(45.4-51.2)
	Lack of knowledge about movement	44.8<br>(42.5-47.2)	43.8<br>(39.7-47.9)	45.3<br>(42.4-48.2)

*P-values ≤ 0.05 were considered significant (shown in bold).
